# A randomized phase 2a efficacy and safety trial of the topical Janus kinase inhibitor tofacitinib in the treatment of chronic plaque psoriasis

**DOI:** 10.1111/bjd.12266

**Published:** 2013-07-08

**Authors:** WC Ports, S Khan, S Lan, M Lamba, C Bolduc, R Bissonnette, K Papp

**Affiliations:** 1 Pfizer Inc.Groton, CT, 06340, U.S.A; 2 The University of MontrealMontreal, QC, Canada; 3 Innovaderm ResearchMontreal, QC, Canada; 4 Probity Medical ResearchWaterloo, ON, Canada; 5 K. Papp Clinical Research Inc.Waterloo, ON, Canada

## Abstract

**Background:**

Tofacitinib (CP-690,550) is a novel Janus kinase inhibitor in development as an oral formulation for the treatment of several inflammatory diseases including psoriasis.

**Objectives:**

This phase 2a study aimed to assess the efficacy, systemic safety, local tolerability and systemic pharmacokinetics of topical tofacitinib in mild-to-moderate plaque psoriasis.

**Methods:**

Two tofacitinib ointment formulations were evaluated in this multicentre, double-blind, vehicle-controlled trial (NCT01246583). Seventy-one patients were randomized 2 : 1 : 2 : 1 to 2% tofacitinib ointment 1, vehicle 1, 2% tofacitinib ointment 2 and vehicle 2, each administered twice daily for 4 weeks to a single fixed 300 cm^2^ treatment area containing a target plaque with or without one or more nontarget plaques and normal skin.

**Results:**

The primary endpoint of percentage change from baseline in the Target Plaque Severity Score at week 4 demonstrated statistically significant improvement for ointment 1 [least squares mean (LSM) –54·4%] vs. vehicle 1 (LSM –41·5%), but not ointment 2 (LSM –24·2%) vs. vehicle 2 (LSM –17·2%). Secondary endpoints (target plaque area and Itch Severity Item) improved similarly for tofacitinib ointment vs. corresponding vehicle. Adverse event (AE) occurrence was similar across treatment groups. All AEs were mild or moderate and none were serious or led to subject discontinuation. One application-site AE (erythema) was reported. Tofacitinib mean systemic exposure was minimal and was greater for ointment 1 than for ointment 2.

**Conclusions:**

Tofacitinib ointment 1 was well tolerated and efficacious compared with vehicle for the treatment of plaque psoriasis. Further study of topical tofacitinib for psoriasis treatment is warranted.

**What's already known about this topic?**

- Janus kinase (JAK) signalling has been implicated in the pathogenesis of psoriasis.
- Tofacitinib (CP-690,550) is a novel, small-molecule JAK inhibitor in development for the treatment of several inflammatory diseases: it has demonstrated efficacy in a phase 2b study in moderate-to-severe plaque psoriasis when given orally.
- Topical therapy is the more commonly used therapeutic option for psoriasis, but there is a need for improved topical treatments.

**What does this study add?**

- In this phase 2a study, tofacitinib in an ointment formulation demonstrated efficacy, systemic safety and local tolerability during 4 weeks of treatment in patients with mild-to-moderate chronic plaque psoriasis.
- Dermal penetration of tofacitinib, a small-molecule, was demonstrated.
- Topical application of tofacitinib has the potential to provide an additional therapeutic option for patients with plaque psoriasis.

Plaque psoriasis is the most common type of psoriasis and is characterized by thickened, erythematosquamous plaques.^1^ First-line management of mild-to-moderate psoriasis involves topical treatment primarily with corticosteroids and vitamin D analogues. Topical corticosteroid use can be limited by local and systemic adverse effects, especially if higher potency corticosteroids are used over the long term.^1^,^2^ Vitamin D analogues are more likely than corticosteroids to cause local skin irritation, and their use can be limited in terms of the application amount and body region. Systemic therapy and phototherapy are used for the treatment of moderate-to-severe disease and are often supplemented with topical therapies.^1^,^2^

Patient dissatisfaction with current topical psoriasis treatments underscores a need for new therapies. There is a particular need for improved topical treatments for patients whose psoriasis is not severe enough to warrant treatment with systemic therapy or whose psoriasis is not adequately controlled with systemic therapy alone.^3^

Tofacitinib (CP-690,550) is a novel, small-molecule Janus kinase (JAK) inhibitor currently in development as an oral formulation for the treatment of several inflammatory diseases including psoriasis. In a cellular setting where JAKs signal in pairs, tofacitinib preferentially inhibits signalling by heterodimers containing JAK3 and/or JAK1 with functional selectivity over receptors that signal via pairs of JAK2.^4^ Tofacitinib inhibits interleukin (IL)-23 signalling by suppression of IL-23 receptor expression, resulting in inhibition of T helper (Th)17 cell differentiation.^5^ Furthermore, inhibition of JAK1 will result in attenuation of signalling by additional proinflammatory cytokines, such as IL-6 and interferon (IFN)-γ,^6^,^7^ as well as type I interferon.^8^ Oral tofacitinib has demonstrated efficacy in a 2-week phase 1 study in psoriasis,^9^ in a 12-week phase 2b study in moderate-to-severe plaque psoriasis^10^ and in other immune-mediated diseases such as rheumatoid arthritis^11^–^17^ and ulcerative colitis.^18^

This is the first reported clinical study of topical tofacitinib ointment therapy for chronic plaque psoriasis. The study compared the efficacy, local tolerability, systemic safety and pharmacokinetics (PK) of two tofacitinib ointment formulations.

## Patients and methods

The study was performed in compliance with the Declaration of Helsinki and the International Conference on Harmonisation Good Clinical Practice Guidelines. The study protocol was approved by the institutional review board or independent ethics committee at each investigational centre, and all patients provided written informed consent.

### Study design and treatment

This phase 2a, randomized, double-blind, parallel-group, vehicle-controlled study (NCT01246583), conducted at 10 centres (four in Canada; six in the U.S.A.), was initiated on 16 February 2011 and completed on 29 November 2011.

Patients were enrolled by the investigators and randomized 2 : 1 : 2 : 1 at baseline using an automated web/telephone randomization system to one of the following treatments: 2% (20 mg g^−1^) tofacitinib ointment 1; vehicle 1; 2% (20 mg g^−1^) tofacitinib ointment 2; or vehicle 2. Randomization occurred across all four treatment groups contemporaneously at all investigator centres. The proprietary ointment formulations contained standard excipients for a topical formulation and differed by one excipient (a penetration enhancer).

Treatments were administered topically twice daily for 4 weeks at a target application coverage of 3 mg cm^−2^ to a single fixed treatment area of 300 cm^2^ [∼1·5% body surface area (BSA)]. The treatment area included one target psoriasis plaque and could contain additional plaques and/or normal skin. On study visit days, showering or bathing, but not moisturizing, was permitted prior to attending. The morning study dose was not applied until instructed. After the final study treatment, the treatment area was left untreated during the 7–10-day follow-up.

### Patients

Eligible patients were aged ≥ 18 years with a diagnosis of stable, chronic, plaque psoriasis for ≥ 6 months prior to the first study dose. Patients were required to have mild-to-moderate plaque psoriasis covering ≤ 10% of their total BSA, and a target plaque area (TPA) ≥ 9 cm^2^ with a Target Plaque Severity Score (TPSS) ≥ 5 and induration subscore ≥ 2.

Exclusion criteria included: nonplaque forms of psoriasis; drug-induced psoriasis or history of psoriatic arthritis; recent systemic or local infection; hepatitis B/C or human immunodeficiency virus infection; history of lymphoproliferative disorder or malignancy, except adequately treated or excised basal/squamous cell carcinoma, or cervical carcinoma *in situ*; evidence of tuberculosis infection; phototherapy within the previous 3 months; treatment with ustekinumab within the previous 12 months or other biologic agents within 6 months; conventional systemic psoriasis treatment within 6 months; or systemic treatments that could affect psoriasis such as oral or injectable corticosteroids, retinoids, methotrexate and ciclosporin within 4 weeks prior to the first study dose.

Topical treatments that could affect psoriasis, e.g. corticosteroids, tars, keratolytics, vitamin D analogues and retinoids, were discontinued for ≥ 2 weeks prior to the first study dose. Exceptions were permitted: hydrocortisone and hydrocortisone acetate ≤ 1% (for use on palms, soles, face and intertriginous areas ≥ 15 cm from the treatment area); tar or salicylic acid preparations or shampoos free of corticosteroids (for the scalp only); and a study-supplied nonmedicated emollient (for all body regions except the treatment area). Potent cytochrome P450 (CYP) 3A4 inhibitors or inducers (Data S1, Table S1; see Supporting information) required washout of ≥ 7 days or five drug half-lives, whichever was longer, prior to the first study dose. Treatment lasting ≤ 7 days with moderate CYP3A4 inhibitors or inducers (except amiodarone) (Table S2; see Supporting information) was permitted during the study. Topical antibacterial and antifungal CYP3A4 inhibitors or inducers were permitted if applied ≥ 15 cm from the treatment area.

### Assessments

The primary comparisons of interest were between the two active treatment groups and their corresponding vehicle groups. The primary efficacy endpoint was percentage change from baseline in TPSS at week 4; this was also evaluated as a secondary endpoint at weeks 1, 2 and 3. Other secondary efficacy endpoints were: change from baseline in TPSS subscores and TPA at weeks 1, 2, 3 and 4. Patient-reported outcomes were the Itch Severity Item (ISI) score change from baseline (weeks 1, 2, 3 and 4), and the proportion of patients in each Patient Satisfaction with Study Medication (PSSM) response category (week 4).

For TPSS and TPA evaluation, a single target plaque was selected at baseline. Plaques that were intertriginous or on the hands, feet, neck, face, elbows, knees, below the knees or on the scalp were not eligible as target plaques or included in the treatment area. The treatment area had to be free of infections and other nonpsoriatic skin conditions. Dermatological clinical evaluations were conducted by experienced dermatologists or physicians, and by the same evaluator (except in the case of an emergency) for each patient.

For TPSS, the target plaque was assessed separately for induration, scaling and erythema using a 5-point severity scale (0, none; 1, slight; 2, moderate; 3, marked; 4, very marked), and the scores summed to produce the TPSS sum score [13-point scale; maximum (most severe) score 12]. For TPA, the target plaque perimeter was traced at each visit and its size quantified by computerized image analysis (planimetry). For ISI, the worst itching due to psoriasis within the treatment area over the previous 24 h was recorded using a numeric rating scale from 0 (no itching) to 10 (worst possible itching). PSSM evaluated overall patient satisfaction with study treatment at week 4 using a single 7-point questionnaire, with options ranging from ‘very dissatisfied’ to ‘very satisfied’. Target plaque photography was performed for illustrative purposes at only two investigator centres.

Safety assessments included the incidence and severity of adverse events (AEs), local tolerability at the treatment area [application-site AEs, burning/stinging (4-point scale: none, mild, moderate, severe)], clinical laboratory values (chemistry, haematology and lipid panels), electrocardiograms (ECG) and vital signs.

Blood samples were collected at week 4 pre- (0 h) and post-dose (1, 2 and 4–9 h) to determine plasma levels of tofacitinib. PK parameters were calculated using noncompartmental analysis of concentration–time data: area under the plasma concentration–time profile from time zero to 12 h (AUCτ), maximum plasma concentration (*C*_max_) and time to *C*_max_ (*T*_max_). Assuming steady state at week 4, AUCτ for the 12-h dosing interval was calculated by assuming the 12-h concentration to be the same as that measured predose (time zero).

### Statistics

The study sample size of 24 patients receiving tofacitinib ointment and 12 receiving vehicle (2 : 1 ratio) provided 88% power to detect a 30% improvement with tofacitinib ointment vs. 10% improvement with vehicle (power estimated based on a one-sided significance level of 0·10 for a two-sample comparison of normally distributed continuous variables, with a common standard deviation of 22%). Each tofacitinib ointment had a corresponding vehicle, resulting in a total target sample size of 72 (2 : 1 : 2 : 1 ratio across the four groups).

The TPSS primary endpoint and other continuous variables were analysed using a random-effects model for repeated measures. Least squares mean (LSM), standard error (SE) and one-sided 90% upper and lower confidence limits (UCL and LCL) were calculated. For comparisons between active treatment and corresponding vehicle, statistical significance was demonstrated if the 90% UCL was < 0 (designated with an asterisk). For changes from baseline (e.g. TPSS subscores) and other continuous variables, the same statistical methods as for the primary endpoint were used. PK data were summarized using descriptive statistics.

The full analysis set included all patients who were randomized to the study and received one or more dose(s) of study treatment.

## Results

### Patients

**Fig 1:**
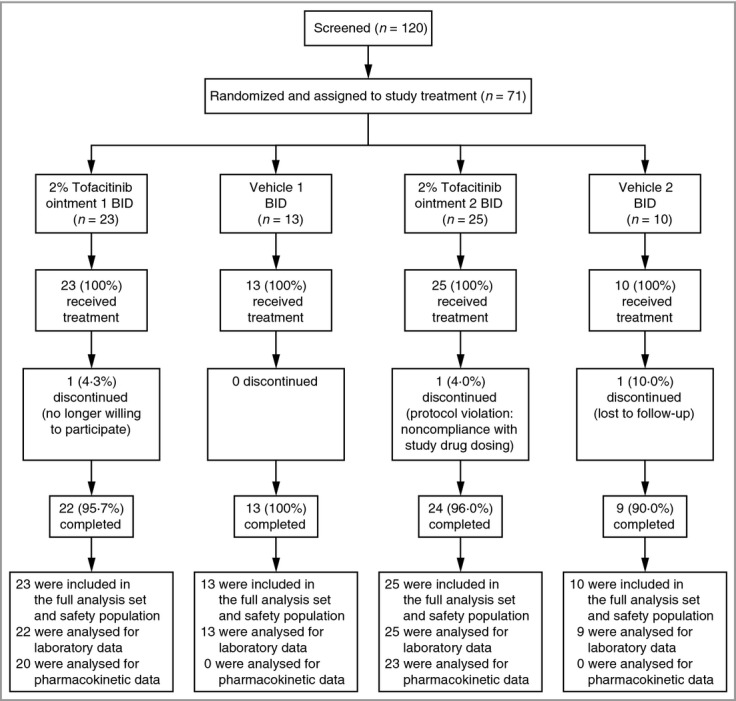
CONSORT diagram. BID, twice daily.

Seventy-one patients were randomized to ointment 1 (*n* = 23), vehicle 1 (*n* = 13), ointment 2 (*n* = 25) and vehicle 2 (*n* = 10), and received treatment (Fig. 1). Baseline demographic characteristics were similar across the groups (Table 1).

**Table 1:** Baseline demography and disease characteristics

	Tofacitinib ointment 1 (*n* = 23)	Vehicle 1 (*n* = 13)	Tofacitinib Ointment 2 (*n* = 25)	Vehicle 2 (*n* = 10)
Sex (male), *n* (%)	17 (73·9)	5 (38·5)	15 (60·0)	6 (60·0)
Age (years), mean (SD)	49·3 (14·5)	50·2 (14·8)	53·8 (14·4)	45·9 (12·6)
Range	24–76	32–72	27–80	26–64
Race, *n* (%)				
White	23 (100·0)	11 (84·6)	24 (96·0)	9 (90·0)
Other	0	2 (15·4)	1 (4·0)	1 (10·0)
Weight (kg), mean (SD)	94·5 (26·0)	85·8 (12·0)	89·2 (17·4)	103·4 (30·3)
Range	58·1–175·0	65·8–104·1	62·2–122·0	61·7–145·2
Body mass index (kg m^−2^), mean (SD)	30·6 (8·6)	30·9 (5·2)	30·6 (6·9)	33·1 (8·8)
Range	22·0–63·1	24·6–40·7	18·6–43·6	22·5–44·8
Duration of psoriasis since first diagnosis^a^ (years), mean (range)	17·1 (0·7–58·4)	19·1 (0·6–51·8)	17·7 (2·5–48·2)	10·5 (2·3–21·5)
PASI, mean (SD)	6·7 (2·51)	6·7 (1·88)	5·9 (2·72)	5·1 (2·93)
Range	3·7–13·5	3·6–10·1	1·5–13·8	2·4–12·4
PGA, mean (SD)	2·45 (0·47)	2·41 (0·43)	2·38 (0·52)	2·47 (0·45)
Range	2–3	2–3	2–3	2–3
BSA^b^ (%), mean (SD)	4·4 (2·13)	5·4 (2·29)	4·1 (2·40)	3·3 (2·78)
Range	0·8–10·0	2·5–9·5	0·9–10·0	0·6–10·0
TPSS,^c^ mean (SD)	7·22 (1·51)	7·31 (1·38)	6·80 (1·19)	7·20 (1·40)
Range	5·0–10·0	5·0–9·0	5·0–9·0	5·0–9·0
TPA^d^ (cm^2^), mean (SD)	34·06 (32·59)	43·73 (31·55)	30·72 (32·60)	44·08 (41·02)
Range	9·3–146·2	9·3–118·3	7·1–142·3	11·4–138·6
ISI,^e^ mean (SD)	4·09 (2·52)	5·54 (3·10)	4·36 (2·53)	6·20 (2·62)
Range	0–9	0–10	0–8	2–10

BSA, body surface area; ISI, Itch Severity Item; PASI, Psoriasis Area and Severity Index; PGA, Physician Global Assessment; TPA, target plaque area; TPSS, Target Plaque Severity Score.

a To day 1 of this study;

b BSA estimated by the handprint method;

c TPSS ranged from 0 to 12 (increments of 1); higher scores represent greater severity of psoriasis;

d TPA was measured by tracing the target plaque perimeter and quantifying its size by computer image analysis (planimetry);

e ISI ranged from 0 (no itching) to 10 (worst possible itching).

### Efficacy

**Fig 2:**
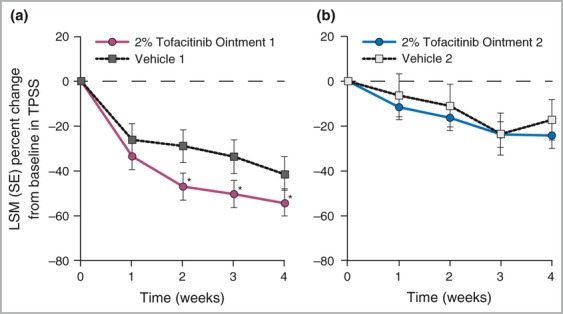
Target Plaque Severity Score (TPSS) percentage change from baseline for (a) tofacitinib ointment 1 vs. vehicle 1 and (b) tofacitinib ointment 2 vs. vehicle 2 (full analysis set, no imputation). *Statistically significant (one-sided 90% upper confidence limit < 0); standard error bars may overlap at the prespecified statistical significance level. LSM, least squares mean.

**Fig 3:**
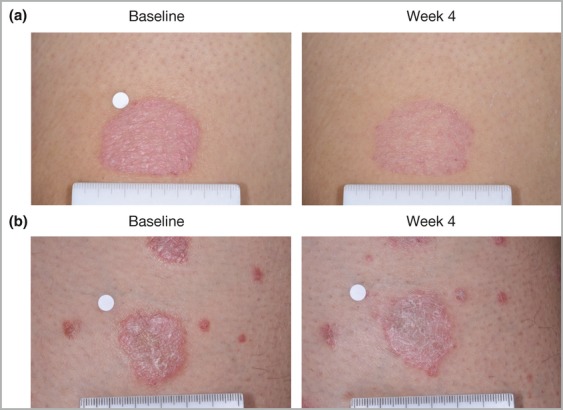
Target plaque photographs at baseline and week 4 for a patient receiving (a) ointment 1 and (b) vehicle 1. Ointment location is left leg above the knee; baseline Target Plaque Severity Score (TPSS) = 8; week 4 TPSS = 3 (–62·5%). Vehicle location is left leg above the knee; baseline TPSS = 7; week 4 TPSS = 6 (–14·3%).

Analysis of the primary endpoint of percentage change from baseline in TPSS at week 4 demonstrated significant differences for ointment 1 (LSM –54·4%) vs. vehicle 1 [LSM –41·5%; difference –12·87; confidence limits (CL) –25·03, –0·71*]; but not ointment 2 (LSM –24·2%) vs. vehicle 2 (LSM –17·2%; difference –6·97; CL –20·57, 6·62) (Fig. 2). Negative values for differences between groups represent a favourable treatment effect. TPSS percentage change from baseline was also significantly different for ointment 1 vs. vehicle 1 at weeks 2 and 3 (90% UCL –5·81* and –4·52*, respectively), but not for ointment 2 vs. vehicle 2 at any time point (Fig. 2). Baseline and week 4 target plaque photographs of patients receiving ointment 1 or vehicle 1 are shown in Figure 3.

**Fig 4:**
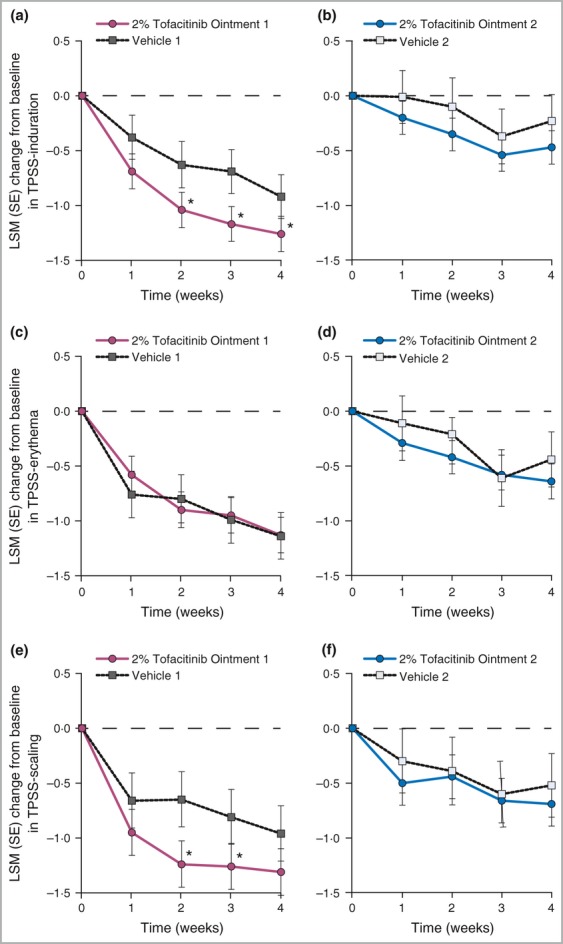
Target Plaque Severity Score (TPSS) – induration, erythema and scaling subscore changes from baseline for tofacitinib ointment 1 vs. vehicle 1 (a, c, e) and ointment 2 vs. vehicle 2 (b, d, f) (full analysis set, no imputation). *Statistically significant (one-sided 90% upper confidence limit < 0); standard error bars may overlap at the prespecified statistical significance level. LSM, least squares mean.

For ointment 1 vs. vehicle 1, the LSM change from baseline in TPSS subscore was significant for induration [weeks 2 (90% UCL –0·08*), 3 (90% UCL –0·15*) and 4 (90% UCL –0·01*); Fig. 4] and scaling [weeks 2 (90% UCL –0·22*) and 3 (90% UCL –0·09*); Fig. 4] but not for erythema (Fig. 4). For ointment 2 vs. vehicle 2, the LSM change from baseline in TPSS subscore was not significant for any subscore at any time point.

The mean TPA values at baseline were higher in the vehicle groups (mean 43·73 cm^2^ and 44·08 cm^2^ for vehicle 1 and vehicle 2, respectively) than in the ointment groups (mean 34·06 cm^2^ and 30·72 cm^2^ for ointment 1 and ointment 2, respectively). All treatment groups had mean percentage decreases from baseline in TPA at weeks 1–4. The LSM percentage change from baseline in TPA was significant for ointment 1 difference from vehicle 1 at weeks 3 (–20·34%) and 4 (–19·04%), but was not significant for ointment 2 difference from vehicle 2 at any time point (Table 2).

**Table 2:** Target plaque area (TPA) least squares mean (LSM) percentage change from baseline (full analysis set, no imputation)

Week	Tofacitinib ointment 1	Vehicle 1	Ointment 1 difference from vehicle 1	Tofacitinib ointment 2	Vehicle 2	Ointment 2 difference from vehicle 2
1	–15·62	–11·06	–4·56 (12·87) [–21·18, 12·06]	–0·90	–3·18	2·28 (14·41) [–16·34, 20·90]
2	–24·16	–10·92	–13·24 (12·73) [–29·69, 3·21]	–4·22	–14·97	10·74 (14·80) [–8·36, 29·85]
3	–33·66	–13·32	–20·34 (12·67) [–36·71, –3·97*]	–2·48	–11·86	9·38 (14·62) [–9·51, 28·27]
4	–38·44	–19·40	–19·04 (12·60) [–35·34, –2·75*]	–8·16	–4·66	–3·50 (14·33) [–22·02, 15·02]

Data are LSM (SE) [upper and lower one-sided 90% confidence limits]. The number of patients with evaluable data in weeks 1–4, respectively, were: 14, 19, 18, 20 (ointment 1); 12, 11, 12, 12 (vehicle 1); 19, 20, 18, 21 (ointment 2); and 8, 6, 7, 8 (vehicle 2). Based on random-effects model for repeated measurement.

* Statistically significant (one-sided 90% upper confidence limit < 0).

The ointment groups had lower baseline mean ISI scores than the vehicle groups; mean ISI scores at baseline ranged from 4·09 in the ointment 1 group to 6·20 in the vehicle 2 group. All groups had mean decreases from baseline at all time points; the mean scores at week 4 ranged from 1·55 in the ointment 1 group to 4·44 in the vehicle 2 group, with the ointment 1 group having the largest LSM decreases from baseline at each time point. At weeks 1 and 4, the LSM change from baseline in ISI was significant for ointment 1 vs. vehicle 1, but not for ointment 2 vs. vehicle 2 at any time point.

Of patients in the ointment 1 group, 50% had PSSM responses of ‘very satisfied’ at week 4, compared with 32% in the ointment 2 group and 23% and 0% in the vehicle 1 and vehicle 2 groups, respectively.

### Safety

Treatment-emergent all-causality AEs were reported for 25/71 (35%) patients; all were mild or moderate. There were no deaths, discontinuations due to AEs or serious AEs. The only AEs to occur in more than one patient were nasopharyngitis (*n* = 4) and urinary tract infection (*n* = 3). One patient treated with ointment 1 reported an application-site AE (erythema); 13 patients (four in the ointment 1 group and three each in the vehicle 1, ointment 2 and vehicle 2 groups) reported predosing burning/stinging at baseline. The number of patients reporting burning/stinging post-dosing at the treatment area was small (*n* = 4 at baseline, and *n* = 5, 5, 2 and 1 at weeks 1–4, respectively) and occurred at a similar frequency across the groups; all reports were mild or moderate, and no patient experienced severe burning/stinging. There were no clinically meaningful median changes from baseline in laboratory, ECG or vital sign parameters across treatment groups. No patient met protocol-defined clinical laboratory safety monitoring or discontinuation criteria during treatment.

### Pharmacokinetics

PK data were available from 43 patients treated with tofacitinib (20 with ointment 1 and 23 with ointment 2). In the ointment 1 group, 12/20 (60%) patients had a systemic concentration at or above the lower limit of quantification (LLOQ; 0·100 ng mL^−1^) for at least one time point, compared with 6/23 (26%) in the ointment 2 group. Following the application of 2% tofacitinib, the median *T*_max_ values were 0·5 and 2 h for ointment 1 and ointment 2, respectively (Table 3). By setting all samples < LLOQ to 0·100 ng mL^−1^, the geometric mean AUC_τ_ in the ointment 1 group (1·92 ng h^−1^ mL^−1^) was found to be approximately 40% higher than in the ointment 2 group (1·38 ng h^−1^ mL^−1^). Similarly, the geometric mean *C*_max_ in the ointment 1 group (0·19 ng mL^−1^) was approximately 55% higher than in the ointment 2 group (0·12 ng mL^−1^).

**Table 3:** Summary of tofacitinib plasma pharmacokinetic parameters after 4 weeks of treatment

	Tofacitinib ointment 1 (*n* = 20)	Tofacitinib ointment 2 (*n* = 23)
*T*_max_ (h)	0·50 (0·00, 4·75)	2·01 (0·00, 3·98)
*C*_max_ (ng mL^−1^)	0·19 (75)	0·12 (47)
AUCτ (ng h^−1^ mL^−1^)	1·92 (61)	1·38 (36)

Data are median (range) for *T*_max_ and geometric mean (%CV) for *C*_max_ and AUC_τ_. For patients with tofacitinib concentrations below the LLOQ, *C*_max_ and AUC were calculated by setting their values to the LLOQ, 0·100 ng mL^−1^. AUCτ, area under the plasma concentration–time profile from time zero to 12 h; *C*_max_, maximum plasma concentration; LLOQ, lower limit of quantitation; *T*_max_, time to *C*_max_.

## Discussion

In psoriatic skin, dermal dendritic cells acquire an inflammatory phenotype and produce cytokines that result in the activation of Th1 and Th17 T cells.^19^ Differentiation of T cells to Th17 cells is supported by IL-6 and IL-21.^20^,^21^ Active Th1 lymphocytes, characterized by tumour necrosis factor and IFN-γ secretion and IL-17-secreting Th17 cells, stimulate the activation and proliferation of epidermal keratinocytes and further production of proinflammatory cytokines and chemokines, thus contributing to the clinical features of psoriasis.^22^ Consequently, the immunomodulatory mechanisms of JAK1 and JAK3 inhibition by tofacitinib are expected to block or attenuate T-cell function, T-cell differentiation and cytokine signalling (e.g. IL-6, IL-21, IL-23, IFN-γ), which play a key role in the pathogenesis of psoriasis.

Results of clinical trials evaluating two JAK inhibitor investigational drugs for the treatment of plaque psoriasis suggests that JAK inhibition can improve psoriasis. In a phase 2b study, 12 weeks of treatment with an oral formulation of tofacitinib resulted in clinical improvement compared with placebo in patients with moderate-to-severe plaque psoriasis.^10^ In a proof-of-concept study with the JAK1/2 inhibitor INCB018424 applied topically in a cream formulation for 4 weeks, the mean total lesion score (scale of 0–4 for erythema, scaling and thickness; total score range 0–12) at week 4 decreased from baseline by 32% with vehicle daily or twice daily; 53% with 1.0% INCB018424 daily; and 54% with 1.5% INCB018424 twice daily. Topical application of INCB018424 was also well tolerated.^23^

This phase 2a study of tofacitinib ointment for the topical treatment of plaque psoriasis met its primary endpoint of percentage change from baseline in TPSS for ointment 1 vs. vehicle 1 (LSM –54.4% vs. –41.5%, respectively); however, the difference between ointment 2 and vehicle 2 (LSM –24.2% vs. –17.2%, respectively) did not achieve statistical significance. TPSS induration and scaling subscores (but not erythema) and TPA secondary endpoints supported these results. The improvement in ISI observed for ointment 1 with the treatment of only a small area of psoriatic skin indicates that the treatment provided significant relief of pruritus and was well tolerated. Both ointment formulations demonstrated local tolerability similar to vehicle, and no clinically significant systemic or local safety signals for tofacitinib were identified.

The percentage decrease from baseline in TPSS observed at week 4 for ointment 1 (–54.4%) is within the range for some other psoriasis topical treatments reporting week-4 efficacy with a similarly described 13-point scale total lesion sum score: vitamin D analogues –32% to –56%^24^–^26^ betamethasone dipropionate –41%;^24^ and a combination of these treatments –61%.^24^

Dermal penetration of tofacitinib was demonstrated by measurable, although limited, systemic levels of tofacitinib when applied to a treatment area of approximately 1.5% BSA. Mean systemic exposures from the current study were approximately 40-fold lower than exposures from the lowest dose tested (2 mg twice daily) in a previous study of oral tofacitinib in patients with moderate-to-severe psoriasis. Furthermore, the highest individual AUC for ointment 1 and ointment 2 was more than eightfold lower than the mean systemic exposure from the 2 mg twice daily oral tofacitinib dose (Pfizer, data on file).

This study was limited by its relatively small sample size, and the efficacy endpoints for localized treatment of selective body region plaques may not be reflective of whole-body efficacy endpoints for treatment of all plaques. Clinical response with treatment longer than 4 weeks cannot be predicted, although an efficacy response plateau for ointment 1 is not evident during the 4-week duration of this study.

The observed difference in efficacy between vehicle 1 and vehicle 2 was unexpected given the only difference between the vehicle formulations was the penetration enhancer contained in vehicle 1. Differences in the vehicles’ emollient properties may explain this difference; however, such speculation should be made with caution due to the small sample size of the vehicle treatment groups (vehicle 1 *n* = 13 vs. vehicle 2 *n* = 10).

In conclusion, this small phase 2a study demonstrated that topical treatment with the JAK inhibitor tofacitinib in an ointment formulation provided improvement in the clinical signs of psoriasis for patients with mild-to-moderate chronic plaque psoriasis. Further study of topical tofacitinib is needed to investigate longer treatment duration, tofacitinib dose and regimen, increased BSA, and use on other body regions (i.e. face, intertriginous areas, elbows and knees) in a larger patient population.

## Study investigators

Canada: Dr Robert Bissonnette, Dr Chantal Bolduc, Dr Wayne D. Carey, Dr Kim Papp and Dr Yves Poulin. U.S.A.: Dr Richard Lee Beasley, Dr Eduardo Humberto Tschen, Dr Amy Markert Morris, Dr Joel Schlessinger, Dr George J Schmieder and Dr Howard Lee Sofen.

## Appendix

W.C.P., S.L. and M.L. are employees of Pfizer Inc. S.K. is an employee of PharmaNet/i3, who were paid contractors to Pfizer in the development of the manuscript, study design stage and to act as the study clinician. C.B. has been a principal investigator, an advisor or consultant, a scientific officer, member of a scientific advisory board, and a speaker for the following groups: Abbott, Amgen, Astellas, Celgene, Centocor-Ortho Biotech, Incyte, Isotechnika, Janssen, Lilly, Medimmune, Merck, Pfizer Inc., Novartis and Welichem Biotech. R.B. has been a principal investigator, an advisor or consultant, a scientific officer, member of a scientific advisory board, and a speaker for the following groups: Abbott, Amgen, Astellas, Celgene, Centocor-Ortho Biotech, Incyte, Isotechnika, Janssen, Lilly, Medimmune, Merck, Pfizer Inc., Novartis and Welichem Biotech. K.A.P. has been a principal investigator, an advisor or consultant, a scientific officer, member of a scientific advisory board, and a speaker for the following groups: Abbott, Amgen, Astellas, Celgene, Centocor-Ortho Biotech, Incyte, Isotechnika, Janssen, Lilly, Medimmune, Merck, Pfizer Inc., and Novartis.

## Supporting Information

**Data S1.** CYP3A4 inhibitors and inducers.

**Table S1.** List of prohibited potent inhibitors and inducers of CYP3A4.

**Table S2.** List of permitted moderate inhibitors and inducers of CYP3A4.
