# Supplementary material for: A randomized phase 2a efficacy and safety trial of the topical Janus kinase inhibitor tofacitinib in the treatment of chronic plaque psoriasis
Source: Br J Dermatol. 2013 Jul 8;169(1):137–45. doi: 10.1111/bjd.12266 (PMC3761190; doi:10.1111/bjd.12266)
Supplement: Supplementary file 1 [file bjd0169-0137-SD1.doc]

**Supporting Information**

*CYP3A4 Inhibitors and Inducers*

Topical (including skin or mucous membranes) application of antibacterial and antifungal medications that were CYP3A4 inhibitors or inducers was permitted, provided these medications were applied at a distance of at least 15 cm from the margin of the study drug treatment area.

Potent inhibitors and inducers of CYP3A4 (Supporting Table 1) were not permitted in the study except in emergency situations requiring no more than one day of administration.

Patients could be initiated on moderate inhibitors (except amiodarone) and inducers (Supporting Table 2), as required, if the total duration of treatment lasted less than or equal to 7 days.

Amiodarone required discontinuation for at least 290 days (~5 half‑lives) prior to the first dose of study drug.

Consumption of juice from grapefruit, pomelos and Seville oranges was permitted up to 8 ounces (total) in a day. It was recommended to separate the application of study drug and the consumption of these fruits and/or their juice by at least 1 hour.

**Supporting Table 1** List of prohibited potent inhibitors and inducers of CYP3A4

| **Potent CYP3A4 Inhibitors** | **Potent CYP3A4 Inducers** |
| --- | --- |
| HIV antivirals:  indinavir  nelfinavir  ritonavir | barbiturates |
|  | carbamazepine |
|  | efavirenz |
|  | modafinil |
| clarithromycin | nevirapine |
| itraconazole | phenobarbital |
| ketoconazole | phenytoin |
| nefazodone | rifabutin |
|  | rifampin |

CYP, cytochrome P450; HIV, human immunodeficiency virus

**Supporting Table 2** List of permitted moderate inhibitors and inducers of CYP3A4

| **Moderate CYP3A4 Inhibitors** | **Moderate CYP3A4 Inducers** |
| --- | --- |
| HIV antivirals:  atazanavir  delavirdine  saquinavir | St. John's wort |
| amiodarone |
| cimetidine |
| clotrimazole |
| diethyl‑dithiocarbamate |
| diltiazem |
| erythromycin |
| fluconazole |
| fluvoxamine |
| Grapefruit or grapefruit‑related citrus fruits and juices for example:  Seville oranges  pomelos |
| mibefradil |
| mifepristone |
| norfloxacin |
| verapamil |
| voriconazole |

CYP, cytochrome P450; HIV, human immunodeficiency virus
